# DRG-based payment and governance in high-complexity thoracic surgery: implications for efficiency and care delivery

**DOI:** 10.3389/frhs.2026.1845824

**Published:** 2026-05-25

**Authors:** Li Ma, Jianxiong Ma, Xiaojing Yan, Yuqing Chi, Jiayin Wei, Shuang Zhou, Xiaoping Zhou, Ying Hu, Xiaomin Nie, Junhua Pan

**Affiliations:** 1 Department of Oncology, Beijing Chest Hospital, Capital Medical University, Beijing, China; 2 Department of Medical Affairs, Beijing Chest Hospital, Capital Medical University, Beijing, China; 3 Clinical Research Institute, Institute of Advanced Clinical Medicine, Peking University, Beijing, China; 4 Department of Medical Insurance, Beijing Chest Hospital, Capital Medical University, Beijing, China; 5 Department of Performance Management, Beijing Chest Hospital, Capital Medical University, Beijing, China; 6 Department of Financial Management, Beijing Chest Hospital, Capital Medical University, Beijing, China

**Keywords:** diagnosis-related groups (DRGs), health services, health services management, tertiary hospital governance, thoracic surgery

## Abstract

**Background:**

Diagnosis-related group (DRG)-based prospective payment is intended to improve efficiency and control healthcare costs, but its impact on high-complexity surgical services remains uncertain. Thoracic surgery, characterized by a high proportion of complex procedures, provides a useful setting to examine how clinical structure and operational performance evolve under DRG constraints.

**Methods:**

A retrospective longitudinal study was conducted using DRG data from 2023 to 2025 in a tertiary hospital. Indicators included surgical volume, case-mix index (CMI), length of stay (LOS), and resource consumption indices. Interdepartmental comparisons were performed within the homogeneous DRG group EB19, and structural differences across DRG groups were evaluated. All analyses were conducted for descriptive and exploratory purposes.

**Results:**

EB19 accounted for 26.1% of thoracic admissions and showed a high DRG-specific CMI (4.02). Surgical volume increased in two units over the study period, while overall case complexity remained stable, indicating no apparent evidence of case-mix dilution at the aggregated level. Mean LOS declined across units, accompanied by reductions in resource consumption indices; for example, the cost consumption index in one unit decreased from 1.52 in 2023 to 0.97 in 2025. Within the EB19 group, substantial interdepartmental variation was observed despite identical DRG classification, with differences in LOS, cost, and financial performance across units. In addition, different DRG groups contributed differently to financial outcomes. The transition from RE16 (2023–2024) to RN16 and RN18 (2025) coincided with more homogeneous grouping and more interpretable performance patterns.

**Conclusions:**

The findings describe patterns of operational variation and convergence across units, suggesting the potential role of governance-related factors in shaping performance under DRG-based payment, with implications for understanding efficiency and care delivery in high-acuity surgical settings. Given the descriptive nature of the study and the absence of patient-level clinical outcomes, the findings should be interpreted with appropriate caution.

## Highlights

- High-complexity DRG groups remained stable despite increasing surgical volume, indicating no case-mix dilution.
- Substantial performance variation was observed across units within the same DRG category (EB19).
- DRG-based payment was associated with partial convergence toward standardized clinical pathways and reduced interdepartmental variability.
- Refinement of DRG classification (RE16 to RN16/RN18) coincided with improved interpretability of performance outcomes.

## 1 Introduction

Diagnosis-related group (DRG)-based prospective payment systems have been widely implemented as a core strategy to improve efficiency and control healthcare expenditure. Originating from the Medicare prospective payment system in the United States, DRG-based payment shifts reimbursement from cost-based to case-based mechanisms, thereby altering provider incentives toward resource optimization within predefined payment bundles. Contemporary evidence suggests that DRG implementation is associated with reductions in length of stay and moderated growth in hospital expenditure; however, the magnitude and direction of these effects vary across settings. Concerns also remain regarding potential unintended consequences, including risk selection, changes in clinical practice patterns, and possible impacts on care quality (1, 2). These mixed findings highlight the importance of context-specific evaluation.

In recent years, DRG reform has been rapidly implemented in China through the China Healthcare Security Diagnosis-Related Groups (CHS-DRG) system. Unlike earlier payment system, the CHS-DRG system simultaneously emphasizes cost containment and the maintenance of high-quality, high-complexity medical services. In particular, national hospital performance evaluation systems require tertiary hospitals to sustain a high proportion of Grade IV procedures, creating a dual pressure of efficiency improvement and structural complexity (3).

Existing research on DRG-based payment has primarily focused on hospital-level outcomes, such as overall expenditure, length of stay, and average efficiency indicators. While these studies provide important system-level insights, they often overlook discipline-level dynamics and the internal heterogeneity that may persist within hospitals. Recent evidence further suggests that the effects of DRG implementation may vary across institutions, patient groups, and healthcare settings (3–5). These findings underscore the importance of examining variation beyond aggregate indicators. DRG-based payment systems have also been widely adopted as a form of activity-based funding to improve efficiency and resource allocation in hospital care (6).

Thoracic surgery represents a prototypical high-complexity specialty in which these dynamics can be examined in detail. As a discipline characterized by resource-intensive procedures and high clinical risk, thoracic surgery is particularly sensitive to policy incentives and organizational governance structures (7, 8). Moreover, thoracic surgical activity—especially high-complexity procedures—has been widely regarded as an indicator of institutional capacity, reflecting both technical expertise and system-level organization.

Using longitudinal DRG data from 2023 to 2025 in a tertiary hospital, this study aims to describe changes in operational performance, structural adaptation, and governance-related patterns within thoracic surgical services under DRG-based payment. In this study, governance is conceptualized as the coordination of clinical pathways, resource allocation, and organizational management processes under DRG-based payment. Specifically, we examine (1) changes in overall service volume and case-mix complexity across DRG groups, (2) performance heterogeneity at both inter- and intra-DRG levels, with detailed analysis of selected representative DRG groups, particularly EB19, and (3) the impact of DRG classification refinement on the interpretation of performance metrics.

## 2 Methods

### 2.1 Study design and setting

This retrospective observational study was conducted at a tertiary public hospital implementing the China Healthcare Security Diagnosis-Related Groups (CHS-DRG) payment system. During the study period (January 2023 to December 2025), thoracic surgical services were delivered by three clinical units (Unit A, Unit B, and Unit C). Given the limited number of time points (*n* = 3), trend analyses were used solely for descriptive purposes and should not be interpreted as evidence of temporal causality. No formal hypothesis testing was conducted, and all analyses were performed for descriptive and exploratory purposes.

### 2.2 Data sources and variables

Data were extracted from the hospital's DRG management platform and inpatient administrative database. All inpatient thoracic surgical cases during the study period were included.

Variables included DRG classification, case-mix index (CMI), surgical volume, average length of stay (LOS), and cost consumption index. The cost consumption index was defined as a relative measure comparing actual resource utilization to the expected cost under DRG-based standards, reflecting the efficiency of resource use. It was calculated according to the CHS-DRG performance evaluation framework established by the National Healthcare Security Administration of China (NHSA) (9). These indicators were selected as commonly used measures to assess case complexity (CMI), efficiency (LOS), and resource utilization (cost consumption index) under DRG-based payment systems.

### 2.3 Definition of analytical framework

To minimize case-mix heterogeneity, interdepartmental comparisons were primarily conducted within the dominant DRG group EB19 (major pulmonary surgery). Because EB19 cases share the same DRG classification and relative weight, differences across units were described as within-DRG performance variation under a shared classification and relative weight. Although EB19 represents a relatively homogeneous DRG category, residual clinical heterogeneity cannot be completely excluded.

In addition, functional differences across DRG groups were explored by comparing EB19, EB29, and chemotherapy-related groups (RE16/RN16/RN18). The transition from RE16 to RN16 and RN18 was analyzed to assess the impact of DRG classification refinement on performance interpretation.

### 2.4 Statistical analysis

Continuous variables were summarized descriptively using means and standard deviations or medians with interquartile ranges, as appropriate.

Temporal trends in key indicators (surgical volume, CMI, LOS, and resource consumption indices) were assessed using descriptive trend assessment based on annual aggregated data, with calendar year treated as a continuous variable. Given the limited number of time points, statistical inference was considered exploratory, and emphasis was placed on consistent directional patterns. Interdepartmental comparisons within EB19 were performed descriptively to evaluate differences in operational performance under homogeneous DRG conditions. Trend analyses were conducted for descriptive purposes only, and no formal inference regarding temporal trends was intended.

All statistical analyses were conducted using IBM SPSS Statistics (version 26.0; IBM Corp., Armonk, NY, USA) and R software (version 4.3.1; R Foundation for Statistical Computing, Vienna, Austria). Margin rate was calculated as (DRG-based reimbursement minus actual hospitalization cost) divided by DRG-based reimbursement. Financial indicators were derived from hospital administrative and DRG billing data.

## 3 Results

### 3.1 Overall DRG profile and volume dynamics

The distribution of major DRG groups and their structural characteristics are summarized in Table 1. Lung surgery–related DRGs constituted the core component of thoracic surgical activity. EB19 (major pulmonary surgery) accounted for 26.1% of admissions and demonstrated a high DRG-specific case-mix index (CMI 4.02), whereas chemotherapy-related DRGs (RE16/RN16/RN18) comprised a larger proportion of cases but were characterized by substantially lower complexity.

**Table 1:** Distribution of major DRG groups in thoracic surgery (2023–2025).

DRG group	Number of cases	Proportion (%)	Case-mix index (CMI)
EB19 (Major pulmonary surgery)	1794	26.1	4.02
EB29 (Other pulmonary surgery)	609	8.9	2.52
Chemotherapy group (RE16/RN16/RN18)^a^	4478	65.0	0.59

a RE16 (2023–2024, DRG v1.1) and RN16/RN18 (2025, DRG v2.0) represent chemotherapy-related groups under different DRG versions.

**Figure 1:**
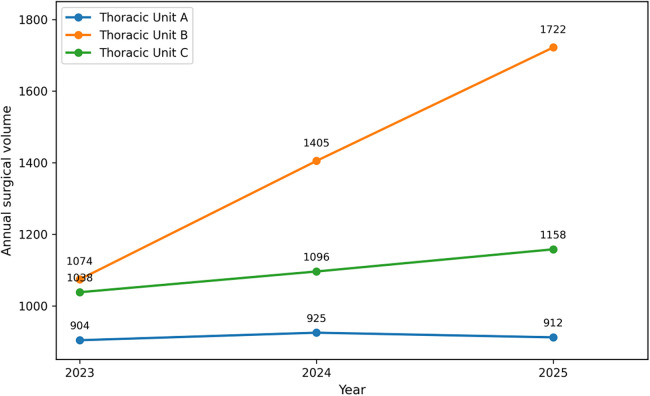
Temporal trends in annual surgical volume across thoracic units from 2023 to 2025.

Between 2023 and 2025, divergent growth trajectories were observed across thoracic units (Figure 1). Thoracic Unit B demonstrated a sustained increase in surgical volume, followed by Unit C, while Unit A remained relatively stable. Importantly, these volume changes occurred without evident reduction in case complexity.

Thoracic Unit B demonstrated a consistent and continuous increase in annual surgical volume over the study period, while Thoracic Unit C also showed an upward trend.

### 3.2 Structural complexity evolution

**Figure 2:**
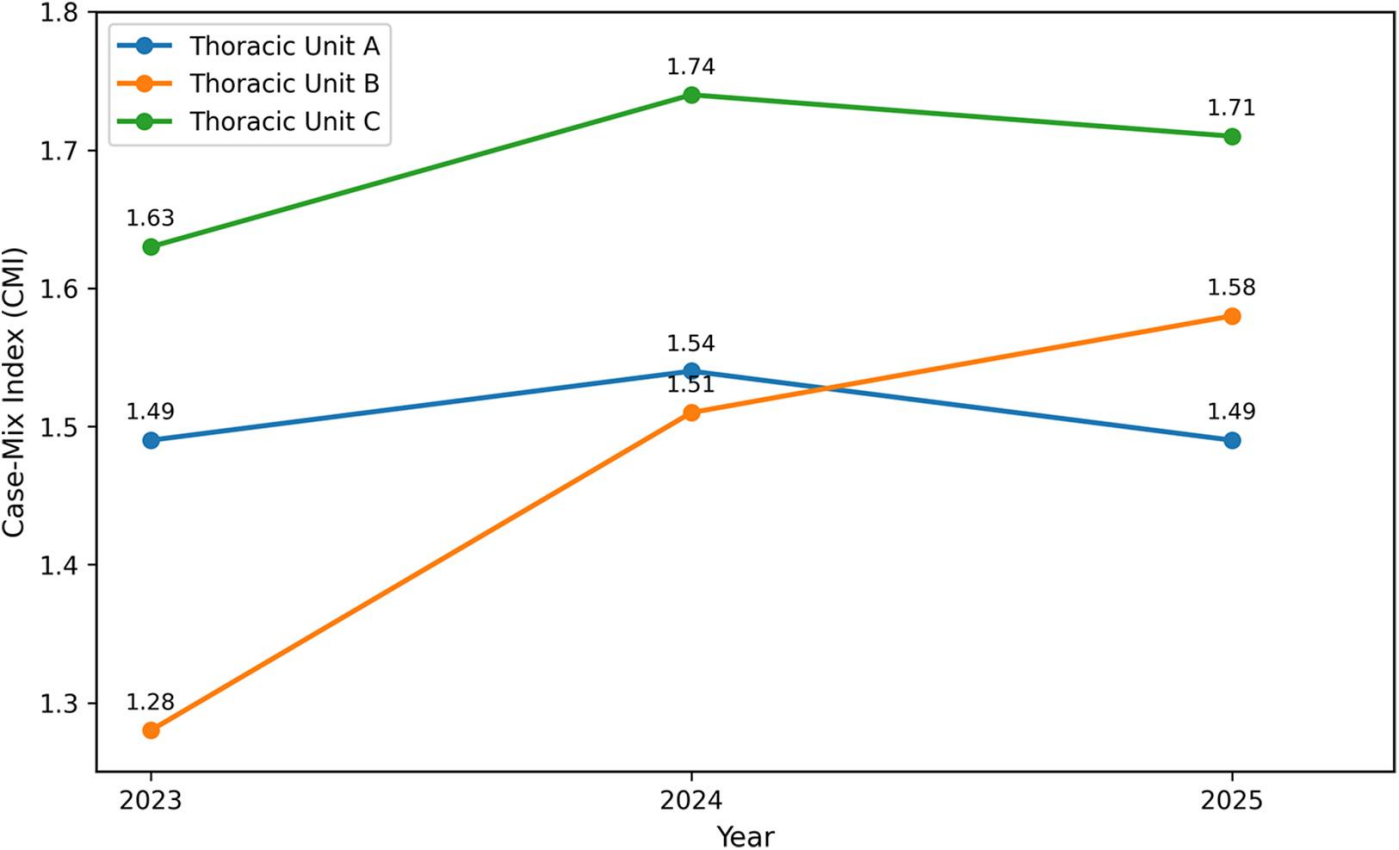
Temporal trends in overall case-mix index (CMI) across thoracic units from 2023 to 2025.

Temporal changes in overall departmental case-mix index (CMI) are shown in Figure 2. Unit C consistently maintained the highest CMI, reflecting a stable high-complexity case profile. Unit B exhibited a consistent upward pattern, showing a pattern consistent with structural upgrading, whereas Unit A remained relatively stable.

Although no statistically significant trend was observed, consistent directional patterns were noted with no observed evidence of case-mix dilution. These patterns are consistent with gradual structural adaptation under DRG-based payment.

Overall CMI remained relatively stable across units, with minor variation observed over time.

### 3.3 Within-DRG heterogeneity: EB19 as a reference group

**Figure 3:**
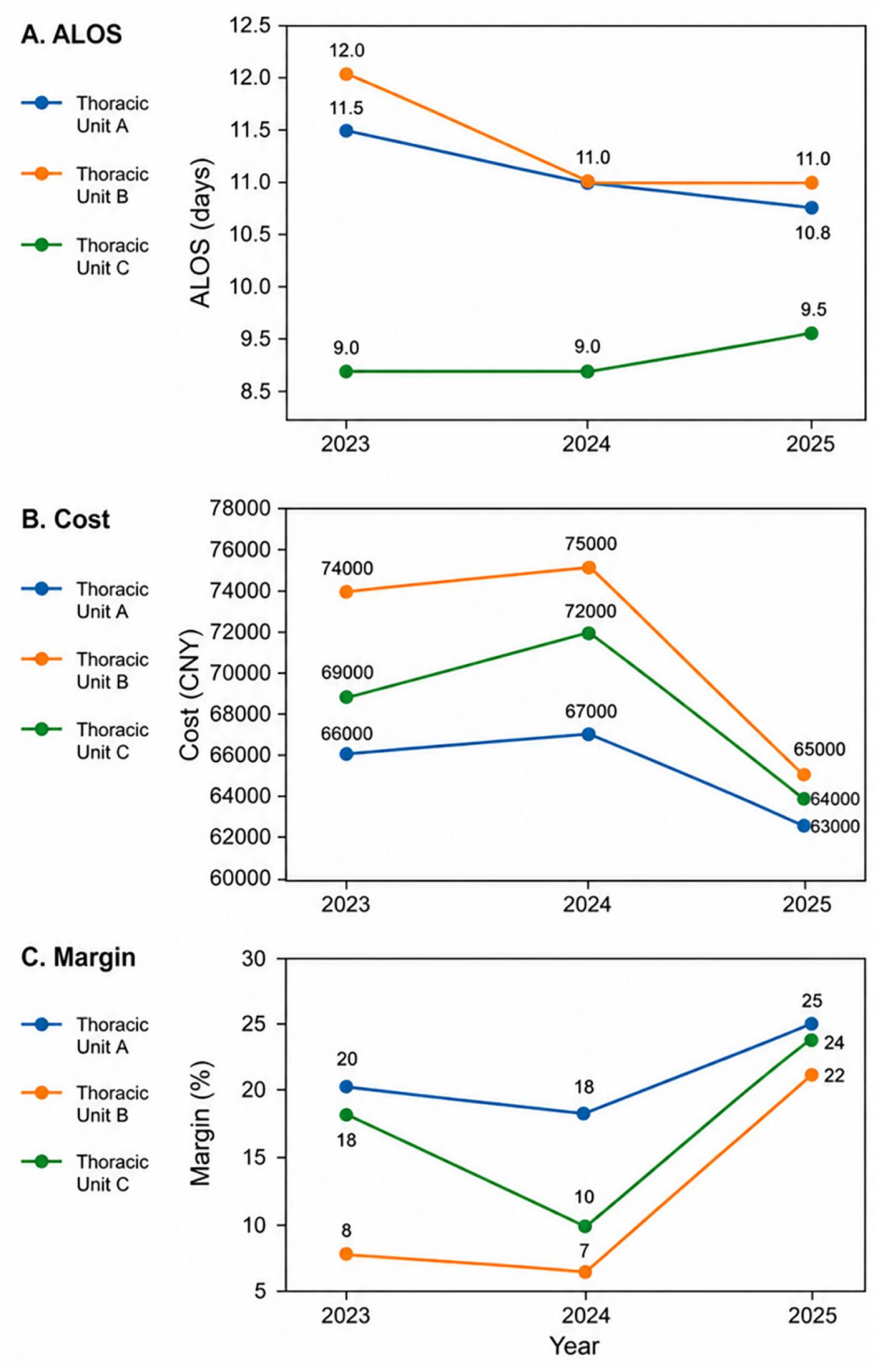
Performance trends of EB19 across thoracic units from 2023 to 2025. **(A)** Average length of stay (ALOS). **(B)** Mean hospitalization cost (CNY). **(C)** Margin rate (%). Unit A, Unit B, and Unit C represent three thoracic surgery units within the same institution. Variation in performance was observed across Units over time.

To minimize case-mix heterogeneity, interdepartmental comparisons were performed within the dominant DRG group EB19 (Figure 3). Despite uniform DRG classification and relative weight, substantial variation was observed across units in terms of length of stay, cost, and financial performance. As shown in Figures 3A–C, interdepartmental differences were consistently observed across average length of stay (Figure 3A), hospitalization cost (Figure 3B), and margin rate (Figure 3C).

Unit C consistently demonstrated shorter hospitalization duration, whereas Unit A achieved higher financial margins. Unit B showed relatively lower performance in earlier years but exhibited notable improvement by 2025. These differences indicate variation in performance across units under the same DRG category.

While Unit C showed shorter length of stay across the study period, Unit A tended to have higher financial margins. Unit B demonstrated relatively lower performance in earlier years, with improvement by 2025.

### 3.4 Functional differences across DRG groups

Beyond EB19, different DRG groups demonstrated distinct functional roles in departmental performance. Compared with EB19, EB29 showed relatively higher financial margins, indicating that DRG groups contribute differently to the overall financial structure of the department.

### 3.5 Classification-sensitive interpretation: transition from RE16 to RN16/RN18

**Figure 4:**
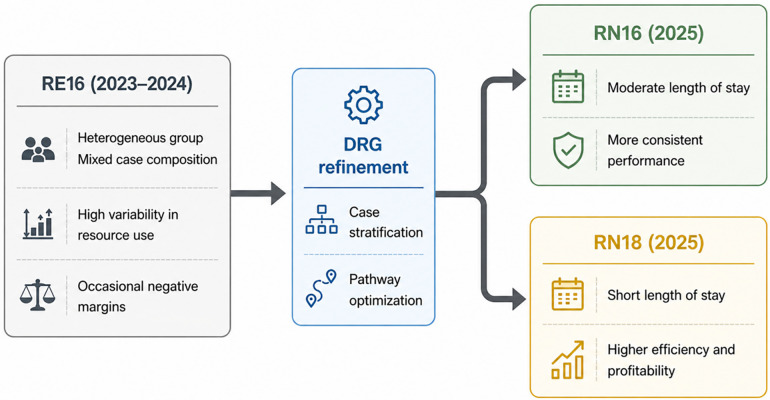
Structural transformation of DRG categories from RE16 to RN16 and RN18.

As illustrated in Figure 4, the transition from RE16 (2023–2024) to RN16 and RN18 (2025) reflects a refinement in DRG classification. RE16 represented a heterogeneous group with mixed case characteristics and substantial variability in performance.

Following DRG version updates, short-stay medical cases were further stratified into RN16 (moderate stay) and RN18 (short stay) with more homogeneous grouping. This structural adjustment allows more interpretable assessment of performance and highlights the importance of considering DRG classification changes when conducting longitudinal analyses. While length of stay decreased across units, no obvious indications of reduced service capacity were observed at the structural level.

RE16 (2023–2024) represented a heterogeneous group with mixed case composition, high variability in resource use, and occasional negative margins. Following DRG refinement, cases were stratified into RN16 and RN18 in 2025. RN16 represents moderate-stay cases with stable financial performance, whereas RN18 represents short-stay cases with high efficiency and profitability. This schematic illustrates the refinement of a heterogeneous DRG category into more homogeneous subgroups, improving interpretability of performance and resource use.

## 4 Discussion

### 4.1 Principal findings

Governance in this study refers to the coordination of clinical pathways, resource allocation, and organizational management under DRG-based payment.

The present analysis describes governance-related patterns in operational performance and structural organization within a high-complexity surgical specialty. Governance is not directly measured; rather, it was interpreted based on observed patterns in operational performance, interdepartmental variation, and convergence over time. These patterns were used as descriptive indicators of governance-related processes rather than direct empirical measures. Across the study period, thoracic surgical services expanded in volume while maintaining case complexity, accompanied by gradual changes in efficiency and resource alignment.

These findings are consistent with evidences showing that changes in efficiency under DRG-based payment often occur alongside pathway standardization and resource optimization, without necessarily indicating reductions in care quality (3, 10, 11). However, most existing evidence has focused on system-level or hospital-wide outcomes, whereas our study provides discipline-level insights within a high-acuity surgical specialty.

Importantly, substantial interdepartmental variation was observed within the same DRG group (EB19), suggesting that performance differences are not solely related to case-mix and may also vary alongside clinical pathways and operational processes. This observation is consistent with recent evidence indicating that variation in clinical pathways, resource utilization strategies, and institutional management approaches may contribute to heterogeneous hospital performance under DRG-based payment systems (2, 12).

The transition from RE16 to RN16 and RN18 helps explain differences in performance interpretation under the updated DRG framework. The RE16 category (2023–2024) represented a relatively heterogeneous DRG group that included cases with varying lengths of stay and resource utilization. In 2025, this category was refined into RN16 and RN18 as part of the updated CHS-DRG classification system. RN16 primarily includes moderate-stay cases with more consistent resource use, whereas RN18 represents shorter-stay cases with lower resource consumption and more stable financial performance. This refinement was intended to improve the clinical homogeneity and resource sensitivity of oncology-related DRG grouping.

From an international perspective, this transition may be understood as a refinement of case grouping to improve comparability across patient subgroups, analogous to approaches used in other DRG-based systems to enhance case-mix homogeneity and performance measurement. This reclassification improves the homogeneity of case groups and facilitates more interpretable performance comparisons. In the present study, this reclassification was associated with improved homogeneity within groups and more interpretable performance patterns, further underscoring the importance of classification-aware analysis. These observations are consistent with recent evidence indicating that refinement of DRG grouping and coding practices can alter measured efficiency indicators, affect comparability across time, and should be explicitly considered in longitudinal analyses of DRG-based payment systems (1, 13).

In addition, different DRG groups contributed unequally to financial performance, with EB29 demonstrating relatively higher margins compared with EB19. This finding highlights the heterogeneous functional roles of DRG groups within departmental financial structures, and is consistent with prior studies showing that DRG-based payment redistributes financial incentives across case categories, resulting in differential revenue and cost profiles among DRG groups (14, 15). This distinction may have potential implications for internal resource allocation and departmental strategy.

Recent evidence suggests that the effects of activity-based funding on hospital performance vary across settings and are shaped by local implementation and institutional adaptation (5, 6, 16, 17).

### 4.2 DRG as a governance tool in international and Chinese context

The role of DRG-based payment observed in this study is consistent with more recent evaluations of prospective payment systems in both international and Chinese contexts. Contemporary evidence indicates that DRG-based reimbursement is often accompanied by reductions in hospital length of stay and moderated growth in per-discharge expenditure. These changes reflect a shift in incentives from volume expansion toward more efficient resource use under fixed payments. However, these effects remain heterogeneous across services, and careful monitoring of care quality is required alongside efficiency gains (18).

In China, recent multi-center and quasi-experimental studies have reported reductions in average length of stay, typically on the order of 1–3 days. Total hospitalization expenditure has also declined in many settings (approximately 10%–15%), with larger reductions observed in selected cohorts (e.g., >20% in some disease groups). These changes have been accompanied by improvements in cost distribution and a reduction in high-cost outliers (3, 19). Disease-specific analyses, including lung cancer populations, have similarly demonstrated concurrent reductions in length of stay and hospitalization costs following DRG implementation, without clear deterioration in short-term clinical outcomes (6, 10).

Our findings are directionally consistent with this body of evidence but provide additional discipline-level granularity. In our cohort, mean length of stay declined across all thoracic units, while resource utilization became more aligned over time; notably, the cost consumption index decreased from 1.52 in 2023 to 0.97 in 2025 in one unit. These changes occurred alongside increasing surgical volume and sustained case complexity (e.g., EB19 CMI 4.02), suggesting that there was no apparent evidence of case-mix dilution in this cohort.

Efficiency-related changes under DRG-based payment should be interpreted alongside care quality considerations (1, 20). Although patient-level quality indicators were not assessed in this study, existing literature suggests that reductions in length of stay and cost under DRG systems are not always accompanied by deterioration in short-term outcomes. Continuous monitoring of clinical outcomes and patient safety indicators remains essential.

Taken together, these findings should be interpreted primarily in relation to efficiency and resource utilization under DRG-based payment. In this context, DRG-based payment may be associated with more standardized and coordinated care delivery, rather than solely reductions in resource use. Our results suggest that, when coupled with effective governance, DRG reform may support more coordinated care delivery in high-complexity surgical services.

### 4.3 Governance heterogeneity within homogeneous DRG groups

A central contribution of this study is the identification of substantial interdepartmental variation within a homogeneous DRG group (EB19). Despite identical DRG classification and relative weight, we observed marked differences across units in length of stay, cost, and financial performance. These interdepartmental differences may reflect variation in clinical pathways and operational practices, suggesting the potential influence of governance-related factors. For instance, one unit consistently achieved shorter hospitalization durations, another realized higher financial margins, and a third demonstrated pronounced improvement over time.

Recent evidence suggests that such variability is common under DRG-based payment systems, with differences in clinical pathways, resource utilization, and institutional practices contributing to heterogeneous performance outcomes even under standardized reimbursement frameworks (1, 20). These findings suggest that DRG-based payment alone does not eliminate variation, but may provide a structured context in which performance differences become more visible.

Our longitudinal data further indicate that this heterogeneity may change over time. Reductions in length of stay, changes in cost indices, and narrowing performance gaps across units suggest partial convergence in operational performance. This transition—from interdepartmental heterogeneity toward greater pathway alignment—may represent evolving coordination in clinical practice and resource use within high-complexity surgical services. This pattern highlights the potential importance of discipline-level governance in translating DRG incentives into more consistent clinical practice.

### 4.4 Structural adaptation and organizational integration

The convergence in performance observed across units was accompanied by organizational changes, including the integration of thoracic surgical services into a unified management structure. This pattern reflects a broader phenomenon reported in recent literature, in which hospitals respond to prospective payment reforms by reorganizing service lines and strengthening internal coordination (21). In this study, governance-related changes were operationalized as observable modifications in clinical pathways, resource allocation patterns, and interdepartmental coordination.

By reducing fragmentation and promoting standardized clinical pathways, organizational integration may support more efficient resource allocation and improve consistency in care delivery (2). In the present study, this process appears to be closely linked to the observed improvements in efficiency and resource alignment. Similar observations have been reported in recent studies suggesting that the effectiveness of DRG-based payment depends on the extent to which institutions implement internal governance and organizational restructuring. However, the absence of a non-DRG comparator or pre-reform baseline limits causal attribution. Observed changes may also reflect secular trends, technological advances, or institutional initiatives independent of DRG implementation.

### 4.5 Policy implications and transferability

Taken together, these findings highlight that DRG-based payment systems can function as governance instruments rather than merely reimbursement mechanisms, a perspective increasingly emphasized in recent literature (22). The transition from interdepartmental heterogeneity toward more unified clinical pathways may reflect an organizational pattern observed alongside efficiency-related changes and performance improvements (2, 3).

From a policy perspective, the results indicate that the effect of DRG reform depends not only on payment design but also on the ability of institutions to implement discipline-level governance strategies, including pathway standardization, internal benchmarking, and organizational integration. Previous studies have similarly reported that DRG implementation may be associated with improved internal coordination, more efficient management processes, and more refined allocation within hospitals (23).

The experience described in this study may provide operational insights for similar tertiary hospitals implementing DRG-based payment. Rather than necessarily constraining high-complexity specialties, DRG-based payment, when accompanied by effective governance, may support more coordinated care delivery and improved resource alignment. However, because patient-level quality outcomes were not assessed and the study was conducted in a single center, implications for broader transferability and value-based healthcare should be interpreted with caution. From a public health perspective, these findings may be relevant to discussions on improving efficiency and standardizing care delivery in high-complexity specialties, although further validation in multi-center settings is needed.

### 4.6 Limitations

This study has several limitations. First, it is based on a single-center design and aggregated data, which limits causal inference. Patient-level outcomes were not included, and the relatively short observation period may limit detection of long-term effects. Given the limited number of time points, the findings should be interpreted as indicative of directional changes rather than formal statistical trends. Second, the absence of patient-level clinical outcomes limits the ability to directly evaluate care quality under DRG-based payment. In addition, the observed changes in efficiency-related indicators may have been influenced by multiple concurrent factors, including technological advancements, evolving clinical practices, and hospital-level management initiatives. Therefore, these patterns should not be attributed solely to DRG-based payment. Third, although this study was conducted in a tertiary hospital implementing the CHS-DRG system, and may share structural and operational characteristics with other high-acuity institutions, variation in institutional capacity, case-mix, and local implementation strategies may limit the applicability of the findings to other settings. Accordingly, the generalizability of these results should be interpreted with caution and further evaluated in multi-center studies. Finally, these findings reflect efficiency and operational patterns within the study context and should not be interpreted as a comprehensive assessment of value-based healthcare. Further research incorporating patient-level clinical outcomes and multi-center data is needed to provide a more comprehensive evaluation.

## 5 Conclusion

Under CHS-DRG reform, high-complexity thoracic surgery maintained structural complexity while demonstrating gradual changes in efficiency and resource alignment. The findings describe a pattern of interdepartmental variation alongside partial convergence in operational performance over time. These observations highlight the potential importance of governance-related factors, including clinical pathway organization and resource coordination, in shaping performance under DRG-based payment. These findings should be interpreted within the scope of efficiency-related indicators and operational performance. Future studies incorporating clinical outcomes and multi-center data are needed to provide a more comprehensive assessment of the impact of DRG-based payment on healthcare performance.

## Data Availability

The raw data supporting the conclusions of this article will be made available by the authors, without undue reservation.
